# Supplementary material for: Recombinant African Swine Fever Virus Arm/07/CBM/c2 Lacking CD2v and A238L Is Attenuated and Protects Pigs against Virulent Korean Paju Strain
Source: Vaccines (Basel). 2022 Nov 23;10(12):1992. doi: 10.3390/vaccines10121992 (PMC9784410; doi:10.3390/vaccines10121992)
Supplement: Supplementary file 1 [file vaccines-10-01992-s001.zip › vaccines-2005844-supplementary.pdf]

Supplementary Materials

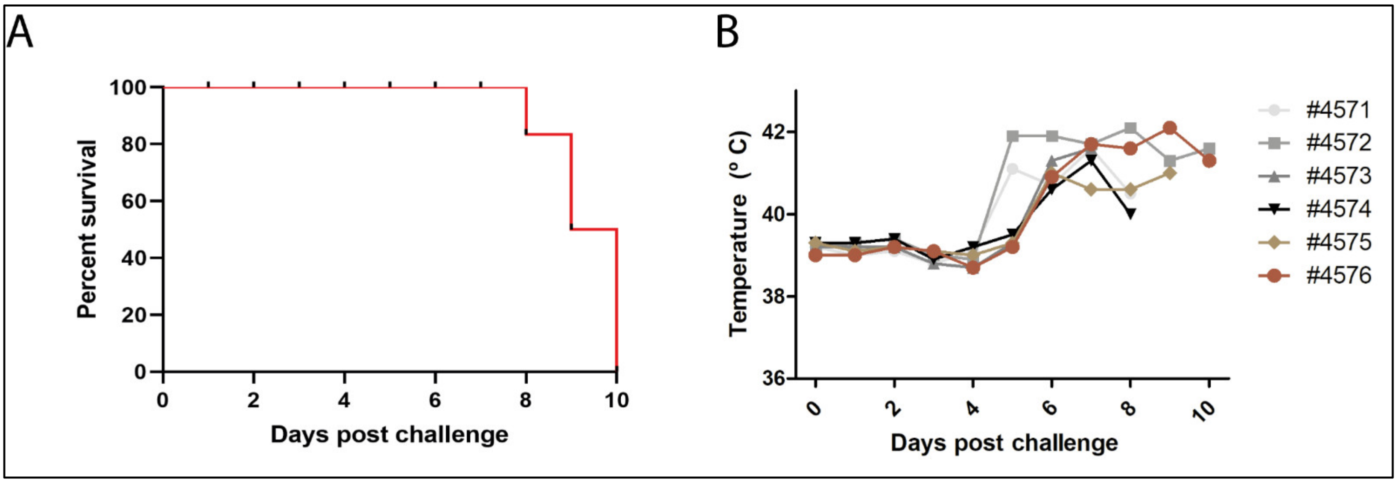

Figure S1. Arm/07/CBM/c2 provoked 100% mortality in inoculated animals. Six pigs (#4571-4576) were inoculated with $3x10^2$ HAD50 of Arm/07/CBM/c2 and observed for 10 days. (**A**) Survival analysis of inoculated animals. (**B**) Daily temperatures of individuals pigs after inoculation.

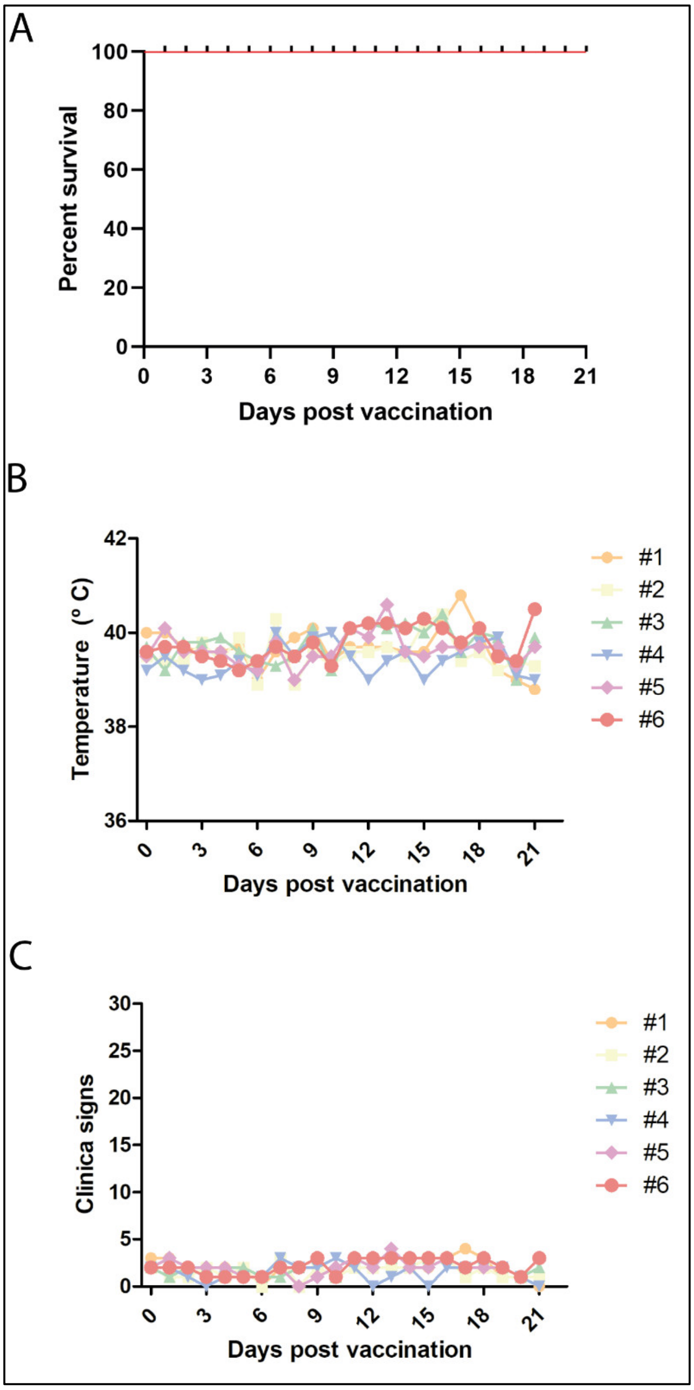

Figure S2. Clinical safety profile study of 5-weeks-old pigs inoculated with the vaccine prototype Arm-ΔCD2v-ΔA238L. (A) Survival analysis of inoculated animals. (B) Daily temperatures of individuals pigs receiving a 10^2 TCID50 dose of the Arm-ΔCD2v-ΔA238L during 21 days after vaccination. (C) Clinical scores of individual pigs receiving a 10^2 TCID50 dose of the Arm-ΔCD2v-ΔA238L during 21 days after vaccination. (Scores were determined based on several parameters, including fever, inappetence, recumbency, skin hemorrhage, hemorrhagic areas on ears and body, joint swelling, laboured breathing and/or coughing, ocular discharge, diarrhea, blood in urine and vomiting (see Table S2)).

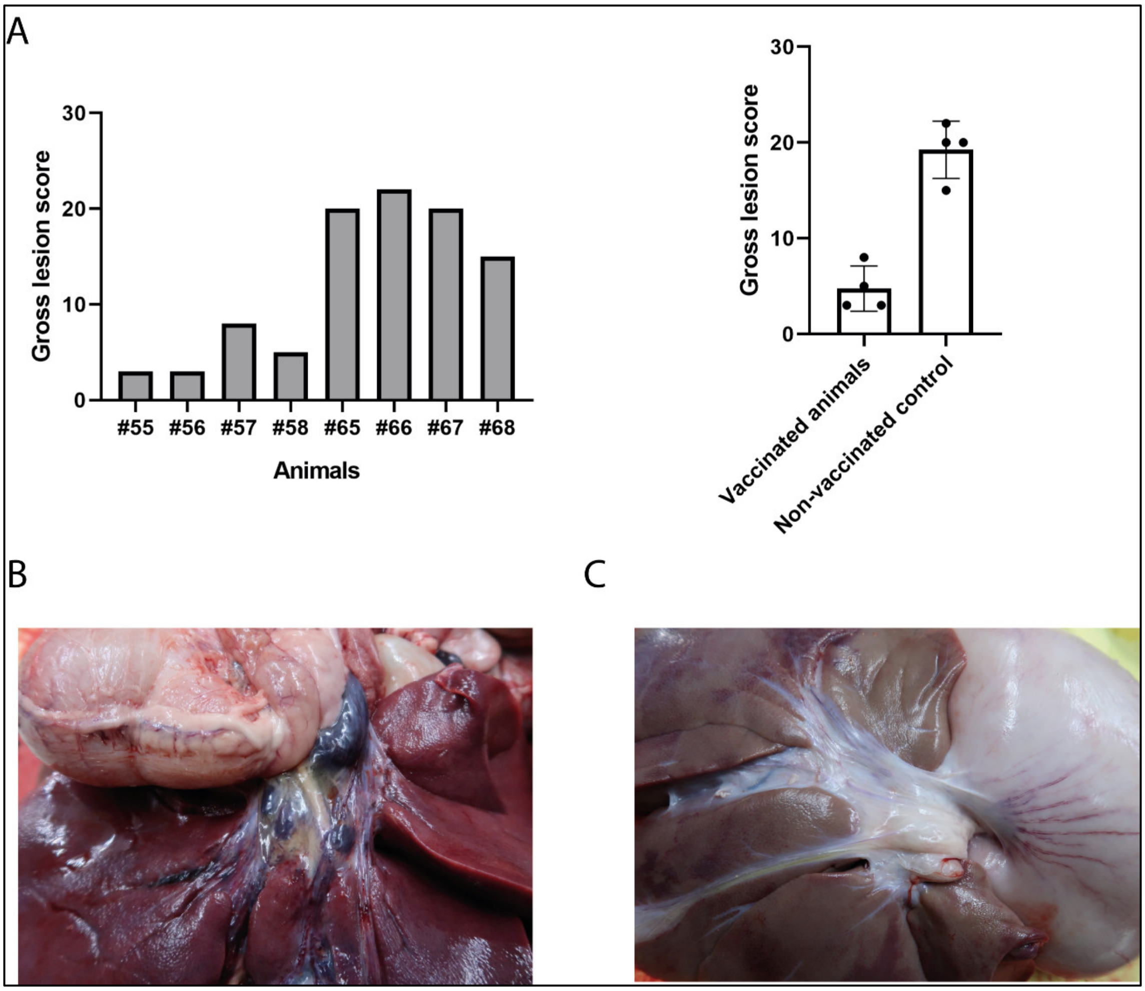

Figure S3. Postmortem histopathological analysis of vaccinated and non-vaccinated animals 21 dpc. (A) Individual (right) or average (left) gross lesion score of pigs receiving a 10^2 pfu dose of Arm-ΔCD2v-ΔA238L, followed after 28 days by challenge with 10^2 HAU of virulent Korean Paju strain (animals #55-58); and non-vaccinated control pigs also challenged with virulent Korean Paju strain (animals #65-68). Gastro-hepatic lymph node of a (B) non-vaccinated and (C) vaccinated animals, after challenge with 10^2 HAU of virulent Korean Paju strain.

Table S1. Numbers of mutations from variant analysis of ASFV WT isolates and recombinant viruses sequencing data.

Position	Gen/Intergenic Region	SNP/Indel	Variant Protein
1163	Intergenic region	A → AC	-
12358	ASFV G ACD 00190	AA → A	Frameshift Leu25STOP
14016	MGF110-14L	A→AC	Frameshift STOP116Val
15444	Intergenic region	AAC → A	-
17403	Intergenic region	C → CG,CGG	-
51229	Intergenic region	T → TGACATTGAT-TATTGAC	-
138874	D205R	G → A	Arg205Gln

Table S2. Clinical signs score index, based on [51].

Temperature	0	<39
	1	39.0 < to < 39.5
	2	39.5 ≤ to < 40
	3	40.0 to ≤ 40.5
	4	40.6 to ≤ 41
	5	<39
Inappetence	0	
	1	Reduced eating
	4	Only picking at food
	6	Not eating
Recumbancy	0	Normal
	1	Lethargic
	2	Get up only when touched
	4	Slow to get up when touched
	6	Remain recumbent when touched
Skin Haemorrhage	0	Normal
	1	Haemorrhagic areas on ears and body
	3	Generalised haemorrhage all over body
Joint Swelling	0	Normal
	1	A joint swelling
	4	Severe swelling with difficulty walking
Laboured breathing and/or coughing	0	Normal
	1	Mild
	3	Severe
Occular discharge	0	Normal
	1	Gummed up eyes
Diarrhea	0	Normal
	1	Diarrhea
	4	Bloody Diarrhoea
Urine	4	Blood urine
Vomiting	4	Vomiting

Table S3. Virus detected by qPCR in nasal swabs samples in vaccinated (#55-58) and non-vaccinated (#65-68) animals at the indicated days post vaccination (dpv) and days post challenge (dpc). Challenge was administrated at 28 dpv. Values indicated Ct. ND= no detection of viral DNA.

	**Vaccinated Animals**				**Non-vaccinated Animals**			
**dpv**	**#55**	**#56**	**#57**	**#58**	**#65**	**#66**	**#67**	**#68**
0	ND	ND	ND	40,00	-	-	-	-
3	ND	ND	ND	ND	-	-	-	-
5	ND	ND	ND	ND	-	-	-	-
7	ND	ND	ND	ND	-	-	-	-
10	ND	ND	ND	ND	-	-	-	-
14	ND	ND	38,17	38,23	-	-	-	-
21	ND	39,57	ND	ND	-	-	-	-
28	ND	ND	ND	ND	-	-	-	-
dpc								
0	ND	ND	ND	ND	ND	ND	ND	ND
3	ND	ND	ND	ND	ND	39,76	ND	ND
5	ND	ND	ND	ND	32,19	32,26	36,46	ND
7	ND	ND	ND	ND	23,97	25,79	26,05	33,12
10	39,78	ND	39,69	39,73	22,59	24,56	27,98	32,37
14	ND	ND	ND	ND			31,44	27,26
21	ND	ND	ND	ND				

Table S4. Virus detected by qPCR in fecal swabs samples in vaccinated (#55-58) and non-vaccinated (#65-68) animals at the indicated days post vaccination (dpv) and days post challenge (dpc). Challenge was administrated at 28 dpv. Values indicated Ct. ND= no detection of viral DNA.

	**Vaccinated Animals**				**Non-vaccinated Animals**			
**dpv**	**#55**	**#56**	**#57**	**#58**	**#65**	**#66**	**#67**	**#68**
0	ND	ND	ND	ND	-	-	-	-
3	ND	ND	ND	ND	-	-	-	-
5	ND	ND	ND	ND	-	-	-	-
7	40,60	ND	ND	ND	-	-	-	-
10	ND	ND	ND	ND	-	-	-	-
14	41,70	ND	ND	39,29	-	-	-	-
21	38,91	ND	39,89	38,45	-	-	-	-
28	ND	ND	40,01	ND	-	-	-	-
dpc								
0	ND	ND	40,01	ND	ND	ND	39,87	ND
3	ND	ND	ND	38,22	32,98	37,27	36,47	ND
5	ND	ND	ND	38,22	29,06	31,82	ND	39,80
7	ND	ND	ND	ND	28,58	29,33	30,18	ND
10	ND	ND	ND	39,15			34,30	30,24
14	ND	ND	38,35	39,07				
21	ND	ND	ND	ND				
